# Swine Disease and Disinfectants

**Published:** 1885-08

**Authors:** 


### Swine Disease and Disinfectants.

Dr. Klein has been experimenting with chlorine as as air-disinfectant, especially in respect to swine-disease, this being easily conveyed by the air. He experimented with two pigs—one healthy, the other diseased—confined in the same stable, and in an atmosphere impregnated with as much chlorine as the animals could endure without evincing discomfort. The healthy pig remained well for so long a time as six hours, for five successive days, provided the air in the compartment was maintained well fumigated with chlorine gas; two good fumigations, up to a marked pungency in the six hours, being required. One good fumigation would effectually disinfect a compartment in which a diseased pig had been.—*Science.*

